# New York Letter

**Published:** 1894-01

**Authors:** Wm. L. Russell

**Affiliations:** 101 West Ninety-third Street; New York


### *CORRESPONDENCE.*

---

### OUR NEW YORK LETTER.

---

NEW YORK, December 15, 1893.

SCARLET FEVER.

At the meeting of the Section on Pædiatrics last evening, Dr. Jacobi gave some useful advice concerning the treatment of this disease. He said that mild cases required few or no medicinal measures. A well ventilated room, with an even temperature of 70° F., oiling of the skin, and a diet of milk, broth, and farinaceous food pretty nearly covered the ground. A dose of calomel at the beginning was desirable, and he thought it a wise precaution against nephritis to keep the patient in bed for several weeks.

There were other cases, however, that tried men's souls, and it was on these that the resources of our art might be well-nigh exhausted. The question of temperature becomes at times an important one. A temperature which would be borne without discomfort after a few days sometimes caused severe nervous symptoms at the beginning. Under such circumstances an antipyretic might be imperative. A warm bath might be sufficient, or a few doses of tincture of aconite, a purging with cool water and alcohol might be required. He did not employ the new antipyretic drugs, nor quinine. If the fever were very high an ice bag to the heart could be used, and a second to the head would assist in allaying any brain symptoms.

The complications of scarlet fever might demand the use of many remedies. He did not agree with those who would discard medicines in acute diseases. He believed that many cases were lost because not enough medicine was given at the proper time. The physician might not need all the agents at his command in any case· Neither did the surgeon all the instruments with which he armed himself, but both ought to be equally commended for having prepared for any emergency.

Among the conditions requiring attention in some cases were those pertaining to the throat. Some inflammation of the throat was present in nearly every case, only those in which infection occurred through a wound, or the generative passages being exempt. When this condition became severe, careful cleansing became necessary. Local applications were usually injurious to young children, and he was accustomed to employ gentle syringing through the nose. Solutions of sodium chloride or boric acid might be sufficient, or weak bichloride of mercury solutions might be necessary. For the phlegmons, which were a very marked feature of some cases, he believed no treatment to be efficient except free incisions. There was seldom much pus, but a large amount of necrotic tissue could be scraped out, and the wound filled with salicylic gauze. Tincture of iodine was the best disinfectant in such cases. It penetrated the tissues instead of forming a coating under which decomposition might continue, as was the case with tincture of chloride of iron. It was not advisable to give an anesthetic as a rule, but he believed that cocaine might be used in weak solutions. He had been surprised to see what extensive operation could be done after injection of the following solutions:

1. Solution of cocaine in water, 1 to 1,000.
2. Solution of sodium chloride in water, 2 to 1,000.

Five parts of number two and one part of number one were mixed, and under injection of this a large tumor was removed.

Incisions into these phlegmons must be made early to be of any advantage.

Ear inflammations which frequently appeared were less dangerous in young subjects, because of the large calibre of the Eustachian tube. Incision of the tympanic membrane was difficult in infants, and seldom necessary. Rheumatic symptoms, which appeared usually during the first week, were best treated as ordinary rheumatism.

Inflammation of the heart should be met by applications of ice, rest and opium. Digitalis should not be given.

Meningitis and other brain disorders might perhaps be prevented by careful attention to the cleanliness of the nose and pharynx.

Nephritis could also be prevented sometimes by prolonged confinement to bed and to the sick room. If uremia appeared, excretion by the skin and intestines should be increased by means of baths and purges. Pilocarpine might be used, but only when the circulation was not markedly enfeebled. When nephritis seemed to be becoming chronic potassium iodide given for a long time was certainly useful.

Dr. Brannan spoke of the success which had met the vigorous efforts at isolation which had been made in Michigan. He referred also to the small number of cases of scarlet fever admitted to the hospital for contagious diseases here, in contrast with the three thousand or four thousand treated at the London Fever Hospital every year. It was, he said, the intention of the Board of Health to compel the removal of more cases, where the conditions at home were not favorable to isolation.

INTESTINAL SURGERY.

At the last meeting of the academy Dr. J. B. Murphy, of Chicago, showed the ingenious button invented by himself for approximating severed intestines, and read an elaborate paper covering the whole subject of intestinal surgery. Dr. Murphy's button is said to be one of the most ingenious and perfect mechanical contrivances ever introduced into operative surgery. It consists of two parts, one of which fits into the other, and is retained in place by a spring, so arranged that the parts can be separated only by a screw movement. The parts of the button are fastened into the ends of the divided intestine by fine sutures, and then brought together, thus bringing the raw surfaces into close approximation. Union takes place without the formation of much scar tissue and the button is discharged from the anus in ten or twelve days. The passage of intestinal contents is provided for by an opening through the center of the button.

Dr. W. W. Keen, of Philadelphia, in discussing the paper, complimented Dr. Murphy on his invention, which, he said, combined the elements of simplicity, speed and security, so desirable in these operations, in a marked degree. He was afraid, however, that sufficient caliber would not be obtained by this method, and that in time a second operation would be necessary. He believed, too, that the escaped button might become an obstructive center itself, especially in the region of the ileo-cecal valve.

Dr. Weir believed that rapidity was highly desirable in these operations, but he felt that Dr. Keen's objections were entirely valid, especially when it was necessary to join stomach and intestine.

Dr. Abbe knew of nothing so good as sutures, by which the best results had been obtained, especially after gunshot injuries.

In reply to these objections Dr. Murphy said that, while they seemed to be valid from a theoretical standpoint, more than a year's experience by several operators had not practically demonstrated them. Pathological investigations had shown, too, that much less scar tissue was caused by accurate end to end approximation by means of the button than by lateral approximation, or the employment of the Lembert suture, which must result in great thickening and danger of stenosis.

He added, that when a diagnosis of intestinal obstruction was made, an operation was indicated. The surgeon should see such cases early, and should be allowed to select his own time for operating. The diagnosis between obstruction and peritonitis might be difficult. It was rendered more so by the use of opium, which he thought ought never to be administered until the case was clear. A circumscribed swelling was diagnostic, and increase of peristalsis indicated obstruction, as in peritonitis peristalsis was diminished. A fatal issue was generally due, in his opinion, either to failure to make a diagnosis or to act promptly after it was made.

### LONGEVITY.

This was the subject considered at the last meeting of the Section on Practice. The discussion was opened by Dr. E. W. Lambert, who said that the question was most difficult to decide in the case of the individual, while for classes, several general rules could be properly applied. No single factor was more important than a good inheritance, though a long-lived father and mother might produce a short-lived progeny, in exceptional instances. The environment in which an individual developed was next in importance; a comfortable, well-protected, and well-organized home, giving the child a start which carried him well into life. The temperament of an individual had much to do with longevity. The placid, philosophical disposition being much more conducive to length of days, than one inclined to irritability and fretfulness. To these might be added temperance in the broad sense. He who was moderate, careful and conservative in all his habits and indulgences, as well as in physical and intellectual pursuits, was in the way of long life. His experience for many years, as medical officer to the Equitable Insurance Company, had taught him that the majority of business men who died of acute diseases were intemperate. Few died of overwork, but many from what they did when not at work. There was no law of temperance applicable to all. Each man could find out for himself what disagreed and what agreed with him, and govern himself accordingly.

Dr. O. H. Roger thought that next to inheritance the most important point was physique. The nearer one approaches the standard weight for his height, the better. He believed that those under weight were less likely to live than those over weight. The latter, however, ought not to have a very large abdomen. He had found on examining into a large number of deaths from tubercular diseases n the New York Life Insurance Company, that seven-tenths of the cases were under the average weight for their heights. He added that the improvement in the average medical practitioner throughout the country during the past five years was very apparent in the reports of insurance examinations. In regard to the matter of height and weight, Dr. Lambert stated that the light weights were likely to die before forty, while the heavy weights would be liable to develop disease after fifty. Two pounds to the inch, was a safe proportion for minimum weight, and the chest should measure at least half the height. The manner of living was certainly most important, especially in the matter of food and drink. An excess of either tended to shorten life, and the constant drinking of alcoholic beverages kept a stomach, which surely required a certain amount of rest, perpetually stimulated.

Dr. Morean Morris regarded the inherited tendencies of a patient as the most important in the treatment of diseases, especially those of a chronic character. Individuals certainly differed much and often unaccountably in the tenacity of life, and of all the factors concerned he believed ancestral inheritance to be most important. Resemblances to ancestors were apparent in outward appearance, and why not in internal constitution. There were fourteen kinds of secretory tissues described by physiologists, and it would seem that on these depended duration of life, more than on any other factor. These resemblances to ancestors could be utilized in determining the probabilities of the individual, and he thought nothing more noteworthy than the size of the head, especially in the basilar region.

The Chairman of the Section, Dr. Quimby, said that proper nutrition would do much to overcome tendencies to degeneration of different organs, and he believed that the point might be studied with advantage in many cases. He had found that many instances of emphysema seen in dispensary cases, were due to lack of nutrition.

WM. L. RUSSELL.

*101 West Ninety-third Street.*

